# Serum anti-glycan-antibodies in relatives of patients with inflammatory bowel disease

**DOI:** 10.1371/journal.pone.0194222

**Published:** 2018-03-29

**Authors:** Florian Kamm, Ulrike Strauch, Frauke Degenhardt, Rocio Lopez, Claudia Kunst, Gerhard Rogler, Andre Franke, Frank Klebl, Florian Rieders

**Affiliations:** 1 Department of Internal Medicine I, University of Regensburg, Regensburg, Germany; 2 Institute of Clinical Molecular Biology, Christian-Albrechts-University of Kiel, Kiel, Germany; 3 Department of Quantitative Health Sciences, Cleveland Clinic Foundation, Cleveland, United States of America; 4 Department of Gastroenterology and Hepatology, University Hospital Zürich, Zürich, Switzerland; 5 Zurich Center for Integrative Human Physiology, University of Zürich, Zürich, Switzerland; 6 Department of Pathobiology, Lerner Research Institute, Cleveland Clinic Foundation, Cleveland, United States of America; University Hospital Llandough, UNITED KINGDOM

## Abstract

**Background:**

Serum anti-glycan antibodies are a promising tool for differential diagnosis, disease stratification and prediction of Crohn’s disease (CD). To investigate possible heritability of the markers we assessed the presence of serum anti-glycan antibodies in affected and unaffected relatives of patients with CD.

**Methods:**

Serum samples of 169 IBD patients of the German inflammatory bowel disease (IBD) network (140 CD & 29 Ulcerative colitis (UC)), 349 relatives of CD patients, 63 relatives of UC patients and 46 healthy controls were tested for the presence of anti-glycan antibodies by ELISA in a blinded fashion. Clinical data of the IBD patients and controls were available.

**Results:**

A higher proportion of non-affected CD relatives was positive for anti-glycan antibodies compared to healthy subjects. No inheritance of a specific pattern of anti-glycan antibodies could be detected. No difference in marker expression depending on the degree of relationship in the non-affected relatives was noted and the presence of family history did not lead to a difference in marker levels in the affected CD subjects.

**Conclusions:**

Non-affected CD relatives had a higher frequency of anti-glycan antibodies compared to healthy subjects. This difference was mild and was found to be true for the overall reactivity to glycan antigens, but not for specific patterns. This may indicate an inherited mechanism resulting in a non-specific increased reactivity to microbial antigens in IBD.

## Introduction

Aside the environment and a dysregulated immune system, genetic factors are a critical component in the pathogenesis of Crohn’s disease (CD) [1]. The heritable nature of this disease has been determined in twin studies and by investigating familial aggregation, with observed concordance rates of 20–40% in monozygotic twins and 0–7% in dizygotic twin pairs, but also by ethnic/racial differences in the prevalence of CD [2, 3]. The host immune response to commensal bacteria is crucial in maintaining mucosal homeostasis. It has become a well-accepted concept that an abnormality of this response is a key contributing factor to disease pathogenesis [1, 4, 5].

In concordance with this notion CD patients exert a measurable immune response to different microbial factors leading to serologic antibodies directed against microbial components [6–8]. Examples for these antibodies are anti-*Saccharomyces cerevisiae* antibodies (ASCA), antibodies against *Pseudomonas*-associated sequence I2 (anti-I2), outer membrane porin C (OmpC) of *Escherichia coli* and against the bacterial flagellin cBir1 (Anti-cBir1) [6, 9, 10]. A different set of serum antibodies directed against microbial antigens are anti-glycan-antibodies, consisting of anti-*Saccharomyces cerevisiae* antibodies (gASCA), anti-mannobioside antibodies (AMCA), anti-laminaribioside antibodies (ALCA), anti-chitobioside antibodies (ACCA), anti-laminarin antibody (Anti-L) and anti-chitin antibody (Anti-C). This set of biomarkers is highly specific for the diagnosis of CD and associated with and predictive of complicated CD courses, signified by the earlier development of stricture, internal penetrating disease or need for surgery [7, 11–15].

The association of the anti-glycan-antibody panel with CD could represent an inherited increased immune response which may be a primary phenomenon or secondary to e.g. increased permeability of the gut [16, 17]. This hypothesis led us to investigate whether there is an increased response to anti-glycan antibodies in affected and unaffected family members of patients with CD.

## Methods

### Study population

#### IBD relatives

IBD relatives, defined as related family members of IBD patients (either CD or UC), were recruited in the year 2007 through the IBD patients from the IBD center of the University Hospital of Regensburg. All relatives of our IBD patients were asked to participate. Only Caucasian families were included in this study. There were between 1 and 8 relatives per reference IBD subject with a median of 2 relatives [25^th^ percentile (P25), 75^th^ percentile (P75): 2, 3]. Family clusters were taken into account for any comparison involving the reference IBD patients vs. their relatives. Of the 349 relatives of CD patients 330 did not have IBD, 13 had CD and 6 had UC. Of the 65 UC relatives 63 were healthy and 2 had UC. The two UC relatives having UC were excluded from this study due to their number being too low for a meaningful analysis. 66.7% of the CD and 74.6% of the UC relatives were first degree relatives (parents, siblings and children) and the remainder more distant relatives (uncles, nieces, grandparents, etc.). The demographic information can be found in **Table 1**.

**Table 1:** Cohort characteristics.

	Reference IBD patients		Relatives of CD patients			Relatives of UC Patients	Healthy Controls
Factor	CD	UC	CD	UC	Non IBD	Non IBD	
	(N = 140)	(N = 29)	(N = 13)	(N = 6)	(N = 330)	(N = 63)	(N = 46)
Female, n (%)	74 (52.9)	12 (41.4)	5 (38.5)	1 (16.7)	176 (53.3)	33 (52.4)	31 (67.4)
Mean age at study, years (SD)	35.8 (13.1)	41.2 (13.9)	34.9 (14.3)	47.4 (21.9)	44.6 (18.3)	47.7 (18.2)	32.5 (7.4)
Mean BMI, kg/m^2^ (SD)	23.0 (4.4)	24.2 (4.0)	23.1 (2.9)	26.0 (4.2)	26.2 (5.3)	26.7 (4.4)	----
Mean age at diagnosis, years (SD)	28.3 (13.0)	34.5 (14.2)	24.8 (9.9)	30.8 (12.4)	----	----	----
Median disease duration, months (P25, P75)	59.7 (10.5, 146.8)	60.9 (36.6, 132.5)	57.8 (28.2, 199.1)	184.6 (111.7, 310.8)	----	----	----
Location, n (%)							
Ileal Involvement	120 (85.7)	----	10 (76.9)	----	----	----	----
Subtotal colitis or Pancolitis	----	20 (71.4)	----	5 (83.3)	----	----	----
Montreal Classification, n (%)							
B1	33 (23.6)	----	5 (38.5)	----	----	----	----
B1p	10 (7.1)	----	2 (15.4)	----	----	----	----
B2	38 (27.1)	----	4 (30.8)	----	----	----	----
B2p	9 (6.4)	----	1 (7.7)	----	----	----	----
B3	25 (17.9)	----	1 (7.7)	----	----	----	----
B3p	25 (17.9)	----	0 (0.0)	----	----	----	----
IBD related surgery, n (%)	99 (70.7)	8 (28.6)	5 (38.5)	1 (16.7)	----	----	----
Relationship to reference IBD patient, n (%)							----
Sister	----	----	2 (15.4)	1 (16.7)	47 (14.2)	8 (12.7)	----
Brother	----	----	3 (23.1)	2 (33.3)	60 (18.2)	8 (12.7)	----
Dicygotic twin	----	----	0 (0.0)	0 (0)	1 (0.3)	0 (0.0)	----
Mother	----	----	2 (15.4)	0 (0.0)	87 (26.4)	16 (25.4)	----
Father	----	----	1 (7.7)	1 (16.7)	61 (18.5)	12 (19.1)	----
Daughter	----	----	1 (7.7)	1 (16.7)	44 (13.3)	10 (15.9)	----
Son	----	----	4 (30.8)	1 (16.7)	30 (9.1)	9 (14.3)	----
First Degree Relative, n (%)	----	----	8 (61.5)	3 (50.0)	222 (67.3)	47 (74.6)	

BMI, body mass index; IBD, inflammatory bowel disease; CD: Crohn's disease; UC: Ulcerative colitis

P25, P75: 25th and 75th percentiles; SD: standard deviation

First Degree relative is mother, father, son, daughter; Second Degree relative is brother and sister

#### IBD and control cohort

The IBD relatives were related to 140 CD and 29 UC patients, which are part of the previously described cross-sectional IBD cohort [11, 18]. An additional set of 46 apparently healthy controls was also investigated. Collection of this cohort occurred between 2000 and 2006. The healthy controls were not related to the IBD patients and had no family history of IBD. The diagnosis of CD and UC was made based on clinical, radiographic, endoscopic and histopathological criteria [19, 20].

### Clinical information

The following demographic data was included: body mass index (BMI), gender, date of sample procurement, age at diagnosis, occurrence of complications, surgery and disease location. Collected data were transferred and stored in a secure coded anonymized database for analysis. The procurement of the clinical data points mentioned above occurred in a blinded fashion to the antibody values. The presence of IBD in the unaffected IBD relatives was excluded by evaluating their history and the absence of typical symptoms. Signed informed consent was obtained from all participants. The ethics committee of the University of Regensburg approved the study.

### Serological analysis

Serum was separated from whole blood by centrifugation and kept frozen at -80°C until use. Serum was analyzed for levels of gASCA IgG, ALCA IgG, ACCA IgA, AMCA IgG, Anti-L IgA and Anti-C IgA in a blinded fashion and as previously described [11, 18]. We used enzyme linked immunosorbent assay (ELISA) following the manufacturer’s protocol (Glycominds, Ltd; Lod, Israel). The cut-off values were chosen based on a previous cross sectional analysis [11]: gASCA 50 ELISA unit (EU), ACCA 90 EU, ALCA 60 EU, AMCA 100 EU, Anti-L 120 EU, Anti-C 50 EU. Samples with measurements exceeding the above cut-off values for the respective antibody were called antibody positive, samples with measurements below the cut-off were called antibody negative.

### Statistical analysis

Descriptive statistics were computed for all clinical variables and the antibody measurements. These include the mean, standard deviation, percentiles for continuous and frequencies for categorical variables. Within each analyzed clinical group we determined the number of samples with 0, 1, 2, 3, 4, 5 or 6 positive antibodies (see above). To assess the overall antibody response within the clinical groups quartile scores for each serological marker were calculated, as described previously [9, 12, 14, 21]. By adding individual quartile scores for each glycan antigen, a semi-quantitative quartile sum score (range 6–24) representing the cumulative quantitative immune response towards all six antigens for each patient was obtained. Comparisons between healthy controls and non-IBD relatives as well as comparisons between reference UC patients and CD relatives with UC were done using Student’s t-tests or the non-parametric Wilcoxon rank sum tests for continuous or ordinal variables and Pearson’s chi-square tests or Fisher's Exact tests for categorical factors. Comparisons between relatives and reference IBD patients were done using generalized linear mixed models with family cluster as a random effect. SAS version 9.2 software (The SAS Institute, Cary, NC) and R version 2.4.1 software (The R Institute for Statistical Computing, Vienna, Austria) were used for all analyses. A *p*<0.05 was considered statistically significant.

## Results

### Status and level of anti-glycan antibodies in CD and UC patients and their relatives

We analyzed the prevalence of anti-glycan antibodies in the reference CD and UC patients (**Table 2**). The measured levels of the anti-glycan markers in the IBD patients was comparable to previously published studies, including our own [11], indicating that no selection bias compared to the whole IBD patient cohort of the University Hospital of Regensburg was present.

**Table 2:** Marker distribution in the different groups.

	Reference IBD patients		Relatives of CD patients			Relatives of UC Patients	Healthy
Factor	CD	UC	CD	UC	Non IBD	Non IBD	
	(N = 140)	(N = 29)	(N = 13)	(N = 6)	(N = 330)	(N = 63)	(N = 46)
Marker levels mean, EU (SD)							
ASCA	89.3 (58.1)	19.9 (13.5)	71.9 (54.5)	60.3 (42.9)	25.2 (24.8)	24.4 (26.2)	21.4 (17.3)
ACCA	61.0 (51.7)	43.6 (28.0)	66.2 (76.4)	85.1 (63.4)	56.0 (55.4)	45.0 (48.8)	35.4 (23.4)^a^
ALCA	46.4 (27.6)	28.6 (20.0)	38.2 (26.5)	32.7 (25.6)	21.4 (16.8)	19.8 (16.8)	31.1 (20.0)^a^^,^^b^
AMCA	79.6 (50.5)	59.6 (42.0)	80.4 (70.4)	35.0 (6.1)†	67.5 (88.5)	55.9 (91.1)	41.2 (17.5)^a^
Anti-L	96.0 (80.1)	48.6 (35.0)	31.7 (19.5)	33.5 (40.9)	20.1 (18.8)	15.4 (13.6)	37.2 (26.2)^a^^,^^b^
Anti-C	43.2 (31.1)	32.8 (22.0)	41.2 (34.4)	34.2 (16.5)	24.4 (23.0)	26.5 (29.1)	22.5 (24.0)
Sum of Quartiles	14.9 (4.7)	10.5 (4.0)	12.6 (3.9)	11.5 (3.3)	9.5 (2.8)	8.7 (2.8)	8.9 (2.7)
Marker positivity, n (%)							
ASCA	89 (63.6)	1 (3.5)	8 (61.5)	3 (50.0)†	38 (11.5)	7 (11.1)	3 (6.5)
ACCA	20 (14.3)	1 (3.5)	2 (15.4)	2 (33.3)	49 (14.9)	4 (6.4)	1 (2.2)^a^
ALCA	42 (30.0)	3 (10.3)	2 (15.4)	1 (16.7)	14 (4.2)	3 (4.8)	3 (6.5)
AMCA	38 (27.1)	1 (3.5)	3 (23.1)	0 (0.0)	41 (12.4)	3 (4.8)	1 (2.2)^a^
Anti-L	41 (29.5)	2 (6.9)	0 (0.0)	0 (0.0)	1 (0.3)	0 (0.0)	0 (0.0)
Anti-C	39 (28.1)	4 (13.8)	4 (30.8)	1 (16.7)	25 (7.6)	5 (7.9)	2 (5.3)
Number positive markers, n (%)							
0	31 (22.1)	20 (69.0)	2 (15.4)	1 (16.7)†	206 (62.4)	49 (77.8)	36 (78.3)^a^
1	31 (22.1)	6 (20.7)	5 (38.5)	4 (66.7)	89 (27.0)	8 (12.7)	10 (21.7)
2	35 (25.0)	3 (10.3)	4 (30.8)	0 (0.0)	29 (8.8)	4 (6.4)	0 (0.0)
3	14 (10.0)	0 (0.0)	2 (15.4)	1 (16.7)	4 (1.2)	2 (3.2)	0 (0.0)
4	22 (15.7)	0 (0.0)	0 (0.0)	0 (0.0)	1 (0.3)	0 (0.0)	0 (0.0)
5	4 (2.9)	0 (0.0)	0 (0.0)	0 (0.0)	1 (0.3)	0 (0.0)	0 (0.0)
6	3 (2.1)	0 (0.0)	0 (0.0)	0 (0.0)	0 (0.0)	0 (0.0)	0 (0.0)

CD: Crohn's disease, UC: Ulcerative colitis, non IBD: non-inflammatory bowel disease affected, EU: ELISA units, SD: Standard deviation, gASCA: anti-Saccharomyces cerevisiae antibodies, ACCA: anti-chitobioside carbohydrate IgA antibodies, ALCA: anti-laminaribioside carbohydrate IgG antibodies, AMCA: anti-mannobioside carbohydrate IgG antibodies, Anti-L: anti-laminarin carbohydrate antibody, Anti-C: anti-chitin carbohydrate antibody

Comparisons between Non IBD relatives and controls were done using chi-square or Fisher's Exact tests for positivity, Wilcoxon rank sum test for number of positive markers and t-tests for marker levels and sum of quartiles.

a: significantly different from Non IBD CD relative

b: significantly different from Non IBD UC relative

Comparisons between reference UC patients and UC relatives of CD were done using chi-square or Fisher's Exact tests for positivity, Wilcoxon rank sum test for # positve markers and t-test for marker levels and sumo of quartiles.

†: significantly different from reference UC patient

We first compared the reference CD patients to their affected relatives: No difference was noted in quality (defined as positive or negative for a certain antibody) or quantity (level of a certain antibody) of the antibodies between CD relatives affected by CD compared to the reference CD subjects. In the CD relatives affected by UC compared to the reference CD subjects only Anti-L levels and the quartile sum score were lower. Interestingly, in the CD relatives affected by UC compared to the reference UC subjects a trend towards higher levels of the glycan markers was detected. More reference UC subjects were negative for all markers compared to the UC affected CD relatives and the frequency of gASCA was higher in the UC affected CD relatives compared to the reference UC patients. This indicates an overall stronger immune response in UC relatives of CD patients compared to UC patients with no CD relatives (**Table 2**).

Secondly, we compared the non-affected CD relatives with their affected CD family members: The levels of gASCA, ALCA, Anti-L and Anti-C, the frequency of gASCA, ALCA and AMCA and the quartile sum score were lower in the non-affected CD relatives. More non-affected CD relatives were negative for all markers compared to the reference CD patients. This confirms that CD patients have higher immune responses to glycan antigens than their non-affected family members (**Table 2**).

Thirdly, the non-affected UC relatives were tested against the reference UC subjects: Non-affected UC relatives had lower levels of ALCA and Anti-L as well as a lower quartile sum score than reference UC subject, while there was no difference in the overall marker frequency or the number of positive markers (**Table 2**).

Fourthly, we compared the apparently healthy controls (no intestinal inflammation or disease, none of the healthy controls were related to each other) to the non-affected IBD relatives: Levels of ACCA, AMCA, ALCA and Anti-L as well as frequency of ACCA and AMCA were lower in the healthy controls compared to the CD relatives. There was a trend towards a higher quartile sum score in the non-affected CD relatives compared to healthy controls. More healthy controls were negative for all markers compared to the non-affected relatives of CD patients (**Table 2**), indicating a more profound immune response towards glycan antigens in the non-affected CD relatives.

### Glycan marker expression by degree of relationship

To further elucidate a possible hereditary pattern of the anti-glycan antibodies, we investigated whether the degree of relationship with the reference CD subject influences the anti-glycan antibody expression. For this purpose, we divided the non-affected relatives of CD patients into first-degree relatives and non-first degree relatives. No difference in anti-glycan antibody expression was noted between these two groups (**S1 Table**), indicating that a relation to a CD patient appears to influence the marker expression, but the degree of relation does not.

### Expression of the glycan markers in CD patients based on family history

Since the degree of relationship did not make a difference in the glycan marker expression in the non-affected CD relatives we investigated whether the presence of a family history in the affected CD subjects influences their immune response towards glycan antigens. For this purpose, we divided the reference CD patients into two groups: CD subjects with IBD relatives and those without. ACCA was the only marker with a higher frequency in the CD subjects that have a family history of IBD compared to no family history of IBD. All other markers, the number of positive markers per patient or the quartile sum score were approximately the same (**S2 Table**).

### Expression levels of the glycan markers in IBD relatives based on the quartile sum score or the ASCA status of the reference IBD population

To assess if the overall strength of the immune response is inherited we tested if the level of the quartile sum score in the reference IBD patients is linked to the quartile sum score (QSS) or the number of positive markers in the non-affected IBD relatives. For this purpose, we divided our IBD reference cohort into two equal numbered parts according the QSS (≥15 or <15). There was no difference in marker expression in the relatives of CD patients with a high versus a low QSS (**Table 3**).

**Table 3:** Marker positivity in the non-affected relatives based on the QSS of the affected reference relative.

Factor	Ref CD relative with QSS ≥ 15 (N = 184)	Ref CD relative with QSS < 15 (N = 146)	p-value
Marker positivity n (%)			
ASCA	20 (10.9)	18 (12.3)	0.68
ACCA	32 (17.4)	17 (11.6)	0.18
ALCA	8 (4.4)	6 (4.1)	0.93
AMCA	23 (12.5)	18 (12.3)	0.97
Anti-L	1 (0.5)	0 (0.0)	0.99
Anti-C	12 (6.5)	13 (8.9)	0.57
Number positive markers			0.74
0	109 (59.2)	97 (66.4)	
1	55 (29.9)	34 (23.3)	
2	19 (10.3)	10 (6.9)	
3	1 (0.5)	3 (2.1)	
4	0 (0.0)	1 (0.7)	
5	0 (0.0)	1 (0.7)	
Sum of Quartiles	9.6 ± 2.8	9.3 ± 2.9	0.35

CD: Crohn's disease; QSS: Quartile Sum Score

gASCA: anti-*Saccharomyces cerevisiae* antibodies, ACCA: anti-chitobioside carbohydrate IgA antibodies, ALCA: anti-laminaribioside carbohydrate IgG antibodies, AMCA: anti-mannobioside carbohydrate IgG antibodies, Anti-L: anti-laminarin carbohydrate antibody, Anti-C: anti-chitin carbohydrate antibody

### Influence of levels of anti-glycan antibodies on the later development of IBD

To assess whether an increased level of anti-glycan antibodies in the relatives of CD patients predisposes to the development of IBD we contacted all relatives 5 years after sample procurement to determine, if they developed IBD. In the UC healthy relatives group information was available on 52 subjects and none of them developed IBD. In the CD healthy relatives group we received 246 responses. A total of 3 patients developed IBD during follow-up (2 CD with an initial QSS of 6 and 9; 1 UC with an initial QSS of 11). The median QSS of the CD relatives remaining healthy was 9 (P25, P75: 7,11).

The underlying data set for this study can be downloaded as **S3 Table**.

## Discussion

We herein show that the frequency of anti-glycan antibodies in non-affected relatives of CD patients is higher compared to healthy controls. The strength of the immune response based on the quartile sum score was not inherited. The degree of relationship did not influence the anti-glycan antibody levels.

This is the to date largest study examining anti-glycan antibodies in relatives of IBD patients. Other serum markers linked to CD have been studied in families, including ASCA and anti-OmpC. In CD ASCA has been shown to be familial in both affected and non-affected relatives of CD patients. This was true for a qualitative (positive or negative for a certain marker) and a quantitative (levels of a certain marker) relationship [22–27]. The prevalence in relatives of ASCA positive CD patients was higher compared to the prevalence in relatives of ASCA negative patients [22], which has been shown in independent cohorts from Europe, USA and Tunisia [26–28]. Mei et al. found comparable results for anti-OmpC [29] with a qualitative and quantitative increase in unaffected relatives of patients with CD. The phenomenon of heritability is not specific for anti-microbial antibodies. Goblet cell antibodies and antibodies against exocrine pancreas are found in increased prevalence in first degree relatives of patients with CD [30, 31]. Interestingly familiarity of ASCA occurs independently of the diagnosis of CD and vertical transmission of the marker from mother to child has been suggested [32]. In CD patients, the presence of a family history was not associated with different levels of ASCA [33]. One report investigated the anti-glycan antibodies ASCA, AMCA, ALCA and ACCA among others in multiple-affected families with CD, but no detailed analysis comparing unaffected relatives of IBD patients and healthy controls was performed [34].

Our study uses a more recent panel of anti-microbial antibodies, so called anti-glycan antibodies, in relatives of IBD patients. We confirmed prior findings of a stronger immune response towards microbial components in non-affected IBD relatives, also with glycans as antigens. This was found to be true for the overall reactivity to glycan antigens. However, the degree of relationship did not make a difference in the expression of the markers, the presence of a family history did not lead to a difference in marker levels in the affected CD subjects and the strength of the immune response was not inherited. These findings argue against a strong inheritable nature of the anti-glycan antibodies in IBD relatives, but may rather present an epiphenomenon due to e.g. increased intestinal permeability, an unidentified inherited risk factor or shared environmental exposures. Nevertheless, they might serve as a risk marker for CD and indicate a family member with an increased risk for developing CD in the future. Our follow-up may have been too short to detect this pattern. A longitudinal study could help clarify this hypothesis.

The present study has several limitations: We did not have access to household members of the affected patients and their relatives as a control population. Therefore, we cannot exclude an environmental contribution to the familial expression of these markers, such as domestic hygiene, diet, other environmental factors or life habits [35]. However, studies of other serum markers, such as ASCA and anti-OmpC [22, 29] suggested that a shared household in adulthood cannot explain the familial aggregation. The low number of non-affected IBD relatives that developed IBD over time does not allow a meaningful analysis of the glycan markers predicting disease development. The detected antibodies may not be specific for the ‘microbial’ glycans, but may be directed against self glycans [36] and this study is not able to answer this definitively. This study is exploratory and no comparison for multiple testing has been performed. The findings in this study need to be externally validated. We do not have information about potential IgA deficieny in our subjects. Three of the anti-glycan antibodies are of the IgA class and this could have influenced the results.

In summary, a higher frequency of non-affected CD relatives was positive for anti-glycan antibodies compared to healthy subjects. This was found to be true for the overall reactivity to glycan antigens, but not for the strength of the response. This could indicate an inherited increased reactivity to microbial antigens in IBD.

## Supporting information

S1 Table (XLS)

S2 Table (XLS)

S3 Table (XLSX)

## Abbreviations

ACCA: Anti-chitobioside carbohydrate antibody
ALCA: Anti-laminaribioside carbohydrate antibody
AMCA: Anti-mannobioside carbohydrate antibody
Anti-C: Anti-chitin
Anti-cBir1: Antibodies against the bacterial flagellin cBir1
Anti-I2: Antibodies against *Pseudomonas*-associated sequence I2
Anti-L: Anti-laminarin
Anti-OmpC: Anti-outer membrane porin C of *Escherichia coli*
BMI: Body mass index
CD: Crohn’s disease
ELISA: Enzyme linked immunosorbent assay
EU: ELISA units
gASCA: Anti-*Saccharomyces cerevisiae* antibody
IBD: Inflammatory bowel disease
UC: Ulcerative colitis
