# Correction: Serum anti-glycan-antibodies in relatives of patients with inflammatory bowel disease

**DOI:** 10.1371/journal.pone.0203709

**Published:** 2018-09-04

**Authors:** Florian Kamm, Ulrike Strauch, Frauke Degenhardt, Rocio Lopez, Claudia Kunst, Gerhard Rogler, Andre Franke, Frank Klebl, Florian Rieder

The ninth author's name is spelled incorrectly. The correct name is: Florian Rieder.
